# Can Ordering Groceries Online Support Diet Quality in Adults Who Live in Low Food Access and Low-Income Environments?

**DOI:** 10.3390/nu15040862

**Published:** 2023-02-08

**Authors:** Daniela C. Avelino, Valerie B. Duffy, Michael Puglisi, Snehaa Ray, Brenda Lituma-Solis, Briana M. Nosal, Matthew Madore, Ock K. Chun

**Affiliations:** 1 Department of Allied Health Sciences, University of Connecticut, Storrs, CT 06269, USA; 2 Department of Nutritional Sciences, University of Connecticut, Storrs, CT 06269, USA

**Keywords:** diet quality, food access, food security, food assistance, poverty, food environment, chronic disease, online grocery ordering, digital literacy

## Abstract

During the COVID-19 pandemic, U.S. food assistance programs allowed the use of program benefits to order groceries online. We examined relationships between the food environment, food assistance, online grocery ordering, and diet quality among adults from one low-income, low food access community in Northeastern Connecticut during the pandemic. Via online survey, adults (n = 276) reported their perceived home and store food environments, food assistance participation, whether they ordered groceries online, and consumption frequency and liking of foods/beverages to calculate diet quality indices. Those who ordered groceries online (44.6%) were more likely to participate in food assistance programs and report greater diet quality. Perceived healthiness of store and home food environments was variable, with the ease of obtaining and selecting unhealthy foods in the neighborhood significantly greater than healthy foods. Healthier perceived home food environments were associated with significantly higher diet qualities, especially among individuals who participated in multiple food assistance programs. Ordering groceries online interacted with multiple measures of the food environment to influence diet quality. Generally, the poorest diet quality was observed among individuals who perceived their store and home food environments as least healthy and who did not order groceries online. Thus, ordering groceries online may support higher diet quality among adults who can use their food assistance for purchasing groceries online and who live in low-income, low-access food environments.

## 1. Introduction

Diet quality, or the concept of the healthfulness of the overall pattern of the diet, is an important determinant of health. In U.S. nationally representative data, poor diet quality is associated with a greater prevalence of chronic disease and/or risk factors such as excess adiposity, elevated blood pressure, and elevated total cholesterol and hemoglobin A1C [1], with low-income households reporting the lowest diet quality and greater incidence of associated disease risk factors [2]. The ability of low-income households to obtain healthy foods is dependent on interactions between the food environment, what is perceived as acceptable, and policies that increase healthy food access and affordability. An improved understanding of the characteristics of the food environment and how these characteristics are perceived is critical for developing effective policies to improve diet quality among low-income communities who also suffer from low access to healthy foods.

Food accessibility is a multidimensional concept, which includes the affordability of healthy foods, distance to a grocery store, the density of grocery stores, and availability of culturally relevant foods [3]. In lower-income neighborhoods, residents may have long travel times to stores with healthy food, and oftentimes, they rely on personal vehicles, which can make transit for grocery trips both time-consuming and expensive [4,5]. With less access to transportation, residents may need to rely on small stores in the neighborhood and face higher prices for many healthy foods and fresh produce due to the lack of supermarkets around them [6,7]. In addition to taste, nutrition, and convenience, the high cost of healthy food affects the purchasing choices of individuals and oftentimes is a major barrier to making healthy food choices [8]. Combining nutrition education with increased access to healthy foods can improve nutrition knowledge and cooking skills to promote healthier diets [9]. Therefore, it would be important to understand the interaction between low-income and healthy food access and their association with the food purchasing and healthy shopping habits of individuals.

An individual’s perception of the food environment is a valid proxy of the availability and accessibility of healthy foods [10] and helps to facilitate an understanding of the impact of the food environment on diet quality and health outcomes. Perceived (respondent-based) measurements may support the limitations of objective measures for evaluating food environments. For instance, objective measures may not accurately reflect individual choices or their overall food access [11]. A number of individual and environmental factors have been linked to food security status, with very low food secure households expressing lower assessments of their neighborhood’s availability, quality, and cost of fruits and vegetables as well as low-fat options [12].

Food insecurity increases the risk of poor diet quality and diet-related chronic conditions such as diabetes [13]. Food-insecure individuals consume fewer fruits and vegetables, which negatively impacts their diet quality and health, and leads to the development of chronic diseases [14]. Food assistance programs target the issue of food and nutrition insecurity in various populations. At the local level, state agencies or nonprofit organizations manage these programs, while the United States Department of Agriculture (USDA) oversees them at the federal level. Government policies and individual factors, including socio-demographic, psychosocial factors, and the perception of the nutrition environment, may modify or mediate the association between the local food environment and eating patterns [10,15]. As the largest supplier of food assistance, the federal government has a substantial capacity to boost access to healthy foods and enhance public health by adequately utilizing nutrition assistance programs like the Supplemental Nutrition Assistance Program (SNAP). Numerous studies have shown that SNAP significantly decreased participants’ food insecurity [16,17].

USDA has expanded the SNAP Online Purchasing Pilot program (OPP) in response to the coronavirus disease 2019 (COVID-19). Through this program, qualified low-income households taking part in SNAP can utilize their monthly government benefits to purchase food from any authorized online grocery store [18]. Since the beginning of the pandemic, online purchasing has steadily been growing, and has become a safe choice for buying groceries with minimal person-to-person contact. According to an observational study of low-income, low-access communities, ordering groceries online has the potential to improve the quality of food purchased for both SNAP participants [19], which may address barriers such as physical access to healthy foods, especially for individuals living in low-income areas that lack access to grocery stores and transportation means [20]. However, SNAP benefits cannot be used to pay for delivery or other related fees [18]. In addition, older adults from low-income and racial/ethnic backgrounds, non-native English speakers, and individuals with disabilities may be disproportionately affected by a lack of access to high-speed internet services or the skills to use digital technology [21]. SNAP benefits may be used differently online in areas with a high percentage of low-income residents and limited access to fresh produce at grocery shops [22]. Since limited access to healthy food is linked to poor diet quality and elevated risks for obesity and cardiovascular diseases [22], more studies are needed to better understand the challenges SNAP recipients face while purchasing food online and its relationship with diet quality [23].

Dietary Quality Indices or Indicators (DQIs) assessed with a variety of methods provide an overall score of diet healthiness as determined by adherence to dietary guidelines and effects on biomarkers that differentiate health status and predict individuals’ risk of chronic diseases [24,25,26]. DQIs are constructed using reported food intake, such as that detailed on food records, recalls, or frequency questionnaires [27,28]. Measures of reported intake are time intensive for participants and researchers and are subject to biases related to memory and social desirability [29]. Diet quality indices also have been constructed from brief dietary assessment instruments [30,31] that are feasible and useful for community-based work. We have found that simply reporting the level of liking and disliking is a reasonable proxy of usual food intake for calculating an index of diet quality that has good psychometric properties, associates with biomarkers of chronic disease, and/or can add precision to dietary assessment and linkages with chronic disease risk [32,33,34,35].

The purpose of the current study is to add to the body of existing literature by examining interactions between online grocery ordering, perception of the food environment, and food assistance participation on measures of diet quality in adults living in a single, low-income, and low access food environment during the COVID-19 pandemic.

## 2. Materials and Methods

### 2.1. Participants

This was an online, cross-sectional study, which recruited a convenience sample of adults who live in a low-income, low food access community in Northeast Connecticut as defined by the USDA’s Food Access Research Atlas food access database [36]. Data were collected between February to April 2022. The study protocol was approved by the University of Connecticut Institutional Review Board (X22-0013).

As part of a collaboration between public health researchers at the university and community partners, we recruited 276 low-income adults from low-income housing complexes and food pantry distribution sites. We distributed study advertisement flyers to mailboxes of the residents living in low-income housing complexes, posted the flyers in the common areas of the low-income housing complexes, and distributed them to the pantry users at food pantry distribution sites within the county. We also distributed the advertisement flyers to potential participants through community-based low-income food and resource agencies. Our inclusion criteria were: (1) adults (≥19 years old), (2) residing in the specific zip code of the community, and (3) participants who speak and read English and/or Spanish. To recruit potential food assistance recipients, we distributed bilingual fliers (English and Spanish) containing a QualtricsXM (Qualtrics, Provo, UT, USA) survey link and a quick response (QR) code and sent emails to low-income housing, food pantries, and community organizations. Participants were also recruited from an active private Facebook group in Connecticut (targeted at the relevant zip codes). Interested participants provided their email addresses and received a unique link.

### 2.2. Procedures

The Qualtrics survey was conducted online on the participants’ personal device (i.e., smart phone, tablet, or laptop computer) with the option of completing the survey in English or Spanish. The online survey was pilot tested for readability and understandability and completion within 25 to 30 min by individuals from the target population.

Participants scanned a QR code or entered a uniform resource locator (URL) website found on a physical or online recruitment flyer to access the survey. Screening questions were used to assess study eligibility, ensuring that individuals were equal to or over 19 years of age and primarily lived in three specific zip code areas. Only those who met the inclusion criteria could proceed. A captcha verification was used to prevent non-human answers, and cookie technology ensured a single submission from each respondent. The participants agreed to participate after reading the information sheet, which was the first question in the online survey. Before rewarding participants with a $25 electronic gift card, researchers examined all survey respondents for consistent responses and checked geolocation to ensure I. P. addresses were in a northeastern region of the U.S.

### 2.3. Sociodemographic, Lifestyle, and Health-Related Characteristics

Demographic information. This information included gender, race/ethnicity, birthplace, primary language spoken, household size, years at current address, educational level, employment status, and marital status.

Health-related questions. Participants were asked to report their body shape using the 9-point Figure Rating Scale [37] for characterization of weight status (1–2 = underweight, 3–4 = normal weight, 5–6 = overweight, and 7–9 = obese), cigarette smoking (current, former, never, including e-cigarettes), cigarette dependence [38], physical activity [39], self-rated health status, self-rated diet quality [40], health insurance status, self-rated overall health status and history of hypertension, diabetes, cancers, and other chronic diseases.

Food Access, Assistance, and Security. Participants were asked about access to digital technology and experience with online grocery ordering. They were asked whether they have access to digital equipment such as a smart phone, tablet, or personal computer; whether they could access the internet at home, housing management office, or at a local public library; whether they have purchased any foods through online grocery ordering; and the barriers for and difficulties with ordering groceries online.

Participants also were characterized by the number of food programs they participated in from a “check all that apply” question, including options for participation in government and non-governmental programs funded programs. The government programs required income verification up to 200% of the U.S. federal poverty guidelines and provided benefits and coupons to purchase food (Supplementation Nutrition Assistance Program, SNAP), including specific foods for pregnant women and children (Special Supplemental Program for Women, Infants, and Children, WIC), or provided nutritious meals at participating in the Child and Adult Care Food Program (CACFP). The non-governmental programs included Feeding America, which oversees several food pantries and mobile food trucks, other not-for-profit organizations offering pantries and meals, and organizations and stores offering food coupons. SNAP participants receive electronic benefit transfer (EBT) cards, which function similarly to debit cards and are reloaded for every month of eligibility. The cards can be used to purchase food items approved by the U.S. Department of Agriculture’s Food and Nutrition Service at participating supermarkets, convenience stores, farmers markets, and online at several retailers.

Food security status was characterized by a two-question screener [41]: “How true is the following statement: Within the past month, we worried whether our food would run out before we could get money to buy more” and “How true is the following statement: Within the past month, the food we bought just didn’t last and we didn’t have money to buy more.” Participants were considered to be food insecure if they often reported worrying about food running out or having an insufficient amount of food.

### 2.4. Perceived Food Environment

Participants completed a modified version of the Perceived Nutrition Environment Measures Survey (NEMS-P) to measure their perceived nutrition environment (community, consumer, home) [10,42]. The NEMS–P has demonstrated good test–retest reliability and acceptable internal consistency [10,42]. Accordingly, availability (whether a large selection of fruits and vegetables is available), affordability (the perception of worth relative to the cost of food items), acceptability (quality of fruits and vegetables), and accessibility (ease of purchasing of fruits and vegetables) can be grouped into the perceived nutrition environment.

### 2.5. Diet Quality Based on Frequency and Liking Responses

Dietary quality was assessed using the Short Healthy Eating Index (sHEI) Survey [31] and the Liking Survey [33,34,35], administered in English or Spanish. The sHEI Survey was developed and validated [31] to assess diet quality with less respondent burden than full food frequency surveys. In a version without food pictures and single questions with example foods or beverages, participants reported the frequency of consumption of fruits, fruit juice, vegetables, green vegetables, starchy vegetables, grains, whole grains, milk, low-fat milk, beans, nuts/seeds, seafood, sugar-sweetened beverages, added sugars, saturated fats, and water. Reported frequencies were compared with the U.S. Dietary Guidelines [43] and then weighted for calculation of the sHEI following published scoring instructions [31].

The Liking Survey was developed and validated [33,34,35] as a simple proxy of usual dietary consumption, fitting the broad taxonomy of dietary behaviors [44]; it is cognitively simple for participants to complete, and rapid to process into a DQI. The Liking Survey has been shown to correlate with multiple measures of self-reported intake [34] and biomarkers of consumption [32,35]. Following the protocol previously reported [45], participants reported their level of liking or disliking of items shown as text with a picture on a line with a slide labeled with 7 faces, ranging from love it, really like it, like it, it’s ok, dislike it, really dislike it, hate it, including never tried or done. The scale score ranges from 100 to −100, with love it/hate it as ±75 to 100, really like/dislike it as ±45 to 75, like/dislike it as ±15 to 45, and it’s ok as 15 to −15. Participants started the Liking Survey by rating their level of liking/disliking of generally liked (receiving a complement, seeing friends and family, going on vacation) and disliked (seeing a mouse at home, being caught in a lie) experiences, and then 3 fruits, vegetables, low-fat protein, fiber, sweets (including sugar-sweetened beverages), high-fat protein, healthy fat, high fat, and refined carbohydrates (Appendix A), as well as exercise (exercising alone, exercising with others, taking the stairs), and sedentary behaviors (watching television, social media). The liking-based DQI was constructed from the average of responses for each food group that was weighted following the Dietary Guidelines 2020 [43]: vegetables (+3), fruits (+2), high-fat foods (−3), fiber foods (+3), sweets (−3), high-fat proteins (−3), low-fat proteins (+3), healthy fats (+2), and refined carbohydrates (−3). Similar to other diet quality indexes, there was a balance of weights for healthy versus unhealthy foods/beverages [35].

### 2.6. Data Analysis

All statistical analyses were conducted using SPSS software (version 28.0, SPSS Inc., Chicago, IL, USA) with a significance criterion of *p* ≤ 0.05 unless indicated. Descriptive analyses were used to describe the participants according to demographic, lifestyle, and health characteristics, with means presented with the number with percentages, standard deviation, or standard error of the mean as indicated. Differences in these characteristics by ordering groceries online (yes/no) or by measures of diet quality (sHEI, liking-based DQI) were assessed by t-test or with the chi-square statistic. Components of the perceived food environment were assessed with exploratory principal component analysis and then with Cronbach’s alpha with the goal of creating internally reliable component scores. Paired *t*-tests were used to compare ratings within an individual for the perceived food environment in a grocery store versus online. One-way ANOVA was used to assess differences between groups that were concordant and discordant for high and low diet quality from the sHEI and liking-based DQI, based on a median split. The equality of variances was evaluated with Levene’s test.

For multivariate analysis to explain variance in diet quality from perceived food environment, food assistance participation, and ordering groceries on measures of diet quality, covariates from the demographic, lifestyle, and health variables were included if they met the significant criterion of *p* ≤ 0.25 in bivariate relationship analyses with measures of diet quality. Model 1 tested components of the perceived food environment on each measure of diet quality, controlling for demographic variables and online ordering of groceries that met the significance criterion. Model 2 added lifestyle and health covariates that met the significance criteria. Model 3 added the interaction term between components of the perceived food environment and food assistance participation (3 levels, none, 1–2, 3 or more). Model 4 added the interactions between components of the perceived food environment and ordering groceries online (2 levels, yes or no). All multivariate analyses were assessed for collinearity by reviewing tolerance and variance inflation factors.

## 3. Results

### 3.1. Participant Characteristics by Online Grocery Ordering

Nearly all the participants (97.4%) came from two zip codes in census tracks characterized by the 2019 USDA food resource atlas [36] as low-income and low food access. According to this atlas, “low-income” areas are where the median family income is ≤80% of the median family income for the state or metropolitan area, and “low-access” are where at least 500 individuals, or 33% of the population, reside further than 1/2 mile (urban areas) or more than 10 miles (rural areas) from the closest supermarket, super- center, or large grocery store. Nearly all (97%) of participants reported access to digital equipment such as smartphones, tablets, personal computers, and internet broadband services.

Table 1 provides the demographic, lifestyle, and health characteristics of the sample by reported online grocery ordering, defined by their responses (yes or no) to the question about purchasing groceries online. Just under one-third (32.2%) of participants who reported receiving food assistance were enrolled in two or more programs. Of the current smokers, nearly half (48.7%) reported high dependence, indicated by responses saying their time to the first cigarette upon waking was ≤30 min. Nearly all (85.5%) participants did not meet the recommended 150 min of physical activity per week, and 25.4% did not perform 30 min of physical activity on any day. At least one chronic health condition was reported by 57.9% of the participants.

Participants reported a mixture of in-store and online ordering (Table 1). Of the total participants, 71% (n = 196) reported physically shopping at the grocery store and carrying out the groceries, whereas just under half (44.6%, n = 123) reported purchasing groceries online. Of those who reported shopping at the grocery store, 26.5% (n = 52) also reported purchasing groceries online. Those who ordered groceries online were more likely to be younger, significantly more men, born in CT, English-speaking, self-report better health, be enrolled in multiple food assistance programs, and smokers (including e-cigarettes). More than half of those reporting receiving federally funded food and nutrition assistance (SNAP, WIC, and CACFP) also reported purchasing groceries online with their EBT card (52.4%).

Among those who relied on ordering groceries online, about one-third had the groceries delivered (~31%), whereas two-thirds picked the groceries up at the store. Of those who did not use their EBT benefits for online ordering (n = 70), about 42% reported a lack of knowledge hindrance (assistance needed to set up an account, use an EBT card to order groceries online, and identify SNAP-eligible food), 33% reported cost barriers (online perceived as more costly, delivery costs not covered by the EBT), and 25% reported a preference for selecting foods themselves (versus having someone else select them).

### 3.2. Perceived Community and Home Food Environment

The sample showed notable variability in the measures of the store, community, and home food environments (Table 2). In alignment with their residence in a low food access community [36], participants averaged between ambivalence and agreeing on perceived quality, availability, and ease of purchasing healthy food in the neighborhood. In paired t-tests, participants reported significantly greater agreement with ease in purchasing sweets and salty snacks versus fruits and vegetables (t = 4.152, *p* < 0.01) and with a large selection of sweets and salty snacks versus fruits and vegetables (t = 6.762, *p* < 0.01). The composite index of healthy food available in the stores was used in the further analysis as it showed good internal reliability and variability (higher score, greater healthy food available) and approximated a normal distribution upon visual inspection. The price of fruits and vegetables did not load onto this composite index and was treated as a separate variable for further analysis, averaging approximately “somewhat expensive”.

Regarding access to stores to purchase foods, most participants reported accessing a store via their own means of private transport, with 72.8% driving their own car, 9.8% accessing public transport, 9.4% walking or biking, and 8% getting a ride. Walking time to travel from their homes and to the store where they typically buy food was evenly distributed between 10-min categories ranging from less than 10 min to over 30 min. Just over one-third (34.1%) of the participants reported their main food shopping venue took more than 30 min. Although 39% of participants who drove their own car were more than 30 min away from their primary food shopping venue, 20% were within walking distance (at least 10 min or less). Factors that influenced the decision on what store participants purchased most of their groceries loaded onto a composite index that was used in further analysis; however, the internal reliability was not strong (alpha = 0.47). Being close to home was the most important factor influencing the store where they purchased most of their food.

For the home food environment (Table 2), the average number of fruits and vegetables in the home within the past week trended toward being greater than the average number of unhealthy foods (4.26 ± 1.86 SD versus (4.04 ± 1.83) (t = 1.77, *p* = 0.08). Participants perceived that they had healthier foods available significantly more frequently than unhealthy foods, averaging between weekly and almost always for fruits and vegetables versus between monthly and weekly for candy and chips (t = 8.98, *p* < 0.01). The composite index of food available at home was used in further analysis as it showed good internal reliability and variability (higher score, greater healthy food available), which approximated a normal distribution upon visual inspection.

### 3.3. Perceived Food Environment by Online Grocery Ordering

Across all participants (n = 276) and paired comparisons within the same participant, the neighborhood versus online ordering was perceived as offering significantly greater quality (3.55 ± 0.97 versus 3.04 ± 1.01, t = 6.84, *p* < 0.01), greater selection (3.71 ± 1.02 versus 3.20 ± 0.99, t = 6.93, *p* < 0.01), and ease in buying fruits and vegetables (3.88 ± 1.00 versus 3.10 ± 1.05, t = 9.85, *p* < 0.01). Interestingly, among those who report ordering groceries online (n = 123), the neighborhood was still reported as significantly greater than online for fruit and vegetable quality (3.5 ± 1.00 versus 3.24 ± 1.07, t = 2.46, *p* < 0.01) and ease of purchasing (3.72 ± 1.08 versus 3.33 ± 1.10, t = 3.25, *p* < 0.01), but no significant difference in product selection was reported (3.59 ± 1.08 versus 3.53 ± 1.10, t = 0.53, *p* < 0.58).

There was no significant mean difference in the measures of perceived food environment (store or home, Table 2) between the adults who ordered groceries online versus those who did not.

### 3.4. Diet Quality Descriptives and Associations with the Food Environment

As shown in Table 3, reported consumption frequencies for food groups and components from the sHEI survey [31] revealed intakes of fruit, vegetables, whole grains, milk, and seafood that fell below the U.S. Dietary Guidelines recommendations [43] in greater than 50% of the participants, and intakes of sugar-sweetened beverages exceeded recommendations in approximately 50% of participants. The sHEI of diet quality showed good variability, ranging from 19.8 to 65.8 and approximated a normal distribution based on visual inspection, with a mean of 42.87 ± 10.32 (95% confidence interval (CI) of 41.64 to 44.09). The sub-scores of the sHEI are shown in Appendix B.

**Figure 1:**
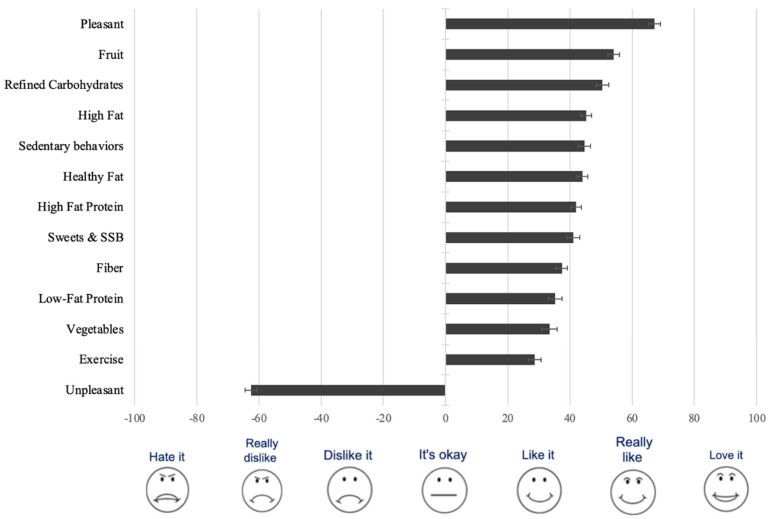
Average reported liking/disliking of foods and activities (±standard error of the mean) ranked most to least in adults (n = 276) from a low-income, low food access community.

Food groups all averaged in the liking range, from the lowest liking for vegetables to the highest liking for fruit; in between disliked unpleasant experiences and liked pleasant experiences. Exercise averaged below the liking for vegetables and sedentary activities averaged near the liking of fatty foods (Figure 1). The liking-based DQI had good psychometric properties. The internal reliability of the groups that made up the index was very good (alpha = 0.843). The index reflected three factors that explained 71% of the variance in all the scores.

Despite the correlation between liking-based DQI and sHEI (β = 0.361, *p* < 0.001), concordant and discordance groups could be identified from the median values for each index. The discordance in these indices was related to the perception of the home food environment (F(3267) = 13.016, <0.001). Individuals with greater liking-based DQI perceived a healthier home food environment regardless of whether they had a higher or lower sHEI (*p*’s <0.001). It appeared the liking-based DQI and sHEI may have influenced the perception of the price of produce (F(3267) = 2.503, = 0.060). In pairwise comparison, individuals with the highest diet quality scores in both indices perceived the price of produce to be significantly greater than those who had the lowest diet quality scores in both indices (*p* < 0.01). The concordant/discordant groups for diet quality did not explain significant differences in the perceived availability of healthy foods in the store.

### 3.5. Bivariate Associations between Participant Characteristics and Diet Quality

Higher diet quality on both sHEI and liking-based DQI was seen in adults who reported being healthier, having a healthier diet, and reported completing 150 min or more of physical activity per week (Table 4). In addition, adults with greater sHEI were more likely to be women, perceive being normal weight, and participate in food assistance programs, whereas greater liking-based DQI was seen in adults whose primary language was not English and who attained higher formal education.

### 3.6. Modeling Associations between Perceived Food Environment and Interactions with Food Assistance Participation and Ordering Groceries Online on Diet Quality

In crude, unadjusted models (Table 5), greater perceived healthiness of the home food environment was associated with significantly greater diet quality scores (both sHEI and liking-based). Additionally, perceived higher price of fruits and vegetables was associated with greater liking-based DQI. There were no significant associations between the perceived availability of healthy foods in stores or between perceived food store access and measures of diet quality. In the models that adjusted for demographic variables (Model 1) and health characteristics (Model 2), the perceived healthiness of the home food environment continued to be associated with significantly greater diet quality scores, whereas the association with the perceived price of fruits and vegetables was slightly attenuated. Reporting yes to ordering groceries was a significant independent predictor of diet quality, both sHEI and liking-based, in Models 1 and 2.

In the models with terms for the interaction between perceived food environment and participation in food assistance programs, adults who perceived greater healthiness of the home food environment and who participated in multiple food assistance programs had significantly greater diet quality scores (both sHEI and liking-based).

The model, including an interaction term for the food environment and participation in food assistance programs, emphasized the importance of perceiving a healthier home food environment on higher diet quality. The highest DQI was observed in adults who reported being on multiple food assistance programs and perceiving the healthiest home food environment. Examining the interaction between food assistance program participation and other indicators of the food environment did not explain further differences in diet quality.

The model, including an interaction term for online grocery ordering and perceived food environment, clarified the ability of the perceived food environment to explain variability in diet quality. The adults who did not order groceries online had the lowest diet quality, especially if they perceived lower quality, selection, and ease of obtaining healthy foods in the store environment. Furthermore, the poorest diet quality was seen in adults who perceived the price of produce as the lowest, especially when using the liking-based DQI. Adults who rated the importance of store location as low in their decision of where to shop for food reported the highest diet quality if they ordered groceries online. Finally, adults who did not order groceries online had the lowest diet quality if they also perceived their home food environment as least healthy.

## 4. Discussion

This online, cross-sectional study explored the association of online grocery ordering on diet quality among individuals from a single low-income and low food access community, highlighting interactions between the perceived food environment, food assistance participation, and online ordering. Our study targeted low-income adults residing in Windham County, CT, since our 2020 assessment of racially/ethnically diverse adult participants of mobile food pantries in this county showed a high prevalence of poverty, food insecurity [46], and chronic diseases (29% diabetes, 49% hypertension, 60% obesity) [47]. Adults who perceived their home and store food environments as less healthy and who did not participate in many food assistance programs or use online grocery ordering had the poorest diet quality. These findings underscore the opportunity to improve online digital and nutrition literacy [21] and increase facilitators and decrease barriers to online ordering [23] with SNAP food assistance programs, especially in unhealthy food environments [3,14,48,49].

The adults in the present study were primarily Hispanic/Latino (37.0%), more than double the frequency compared to the state of Connecticut (17.7%) [50]. However, our sample is closely approximated with the target zip codes for this study, which contain a prominent Hispanic/Latino population (47.8%) [50]; therefore, our sample appears representative. Although there has been significant progress in addressing income inequality in minority groups, there has been an increase in the number of Hispanics living in census tracts with a high level of poverty from 2000 to 2020 [51]. It is important to note that our target sample is from a geographic area with high levels of participation in food assistance, specifically SNAP enrollment in CT, with 21.76% of the population receiving benefits [52]. In addition, 63.8% of our sample reported receiving public health insurance, whereas only 37.1% of CT residents received public health insurance [50]. A greater proportion of survey participants reported receiving federal financial support through health insurance and nutritional assistance programs; 17.4% of participants reported being food insecure, consistent with the insecurity rate across Connecticut, which was estimated to have doubled in 2022 [53]. The self-rated health status of our sample had significantly higher frequencies of fair and poor health and lower frequencies of very good and excellent compared with Connecticut residents who also reported lower incomes (≤30 K to 100 K) [54].

The COVID-19 pandemic increased the risk of food insecurity, particularly among low-income individuals and families [55], with food assistance programs in place to decrease the risk of food insecurity through the provision of benefits for purchasing foods [16,56]. Food assistance extends beyond federal nutrition programs to encompass a network of food banks and pantries organized by Feeding America [57] and private organizations. During the pandemic, there was an increase in food insecurity and food assistance program participation [55], suggesting that individuals had to use a variety of food assistance programs to alleviate food insecurity. Indeed, although previous research reported that federally funded food assistance program participation decreased participants’ food insecurity [16,56], the programs may not provide enough support to enhance diet quality [58] without attention to the food environment. Our results showed a significant association between the perceived food environment, especially the home food environment, and interactions with food assistance participation and ordering groceries online. Higher diet quality was seen in those who perceived their home food environment as healthier. This difference was also found in models adjusted for demographic variables and health characteristics.

In a recent systematic review, perceived healthy food environments were positively associated with healthy dietary habits [59]. However, there is a lack of studies assessing the link between diet quality and perceived food environment [14,60,61,62]. This current study measured diet quality using feasible and valid assessment tools, including a short food frequency survey (sHEI) [31] and a food liking survey [35]. Using both metrics, higher-quality diets were associated with a home food environment. Given the important role of the home food environment in diet composition, focusing on grocery selections and the availability of healthy foods may be a way to enhance diet quality [2]. For instance, in a group of mostly African American, low-income, overweight/obese women, higher consumption of fruits and vegetables was predicted by having more of them in the home [63]. A study examining eleven dimensions of the home food environment showed that all dimensions were significantly associated with meeting national fruit and vegetable guidelines [64].

Perceptions of the cost, the availability of healthful foods, and, to a lesser extent, proximity to a supermarket may influence the consumption of fruits and vegetables [11,62]. Although the cost may be a barrier to meeting fruit and vegetable consumption, the perception of produce affordability has inconsistent associations with diet [11]. Interestingly, in unadjusted models, we found that greater perceived price was associated with better diet quality in the liking-based measure. The pattern was similar for those who ordered groceries online, especially using the liking-based measure. While the price may be a barrier to purchasing fruits and vegetables [65,66], the lower price of these nutritious foods in itself may not compel people to purchase them [67]. Interestingly, store-based audit studies found that more expensive produce pricing was linked to greater consumption of fruits and vegetables [68,69]. A possible explanation may be the quality of produce in the more costly stores was considerably higher, leading to more people favoring the produce. Country-wide differences in the price of fruits and vegetables also do not seem to explain differences in how much they are consumed [70]. Despite intensive policy and community interventions to advance food access equity, the lack of significant improvement in supermarket accessibility in the most vulnerable neighborhoods calls for careful examination and innovative rethinking [51]. Moreover, due to the difficulties in establishing and maintaining a traditional grocery store in low-income, minority communities, online grocery shopping (e-grocery) seems to present a unique opportunity for investigation [51]. Our study showed that adults with the poorest diet quality did not order their groceries online, mainly if they considered healthy foods harder to find and of inferior quality in-store environments. Community-based efforts are required to understand the complex interaction between food availability, quality, and price to improve consumption of healthier diets in diverse, low income communities [70].

Online grocery shopping has received considerable attention since the beginning of the COVID pandemic, and there has been an increased focus on determining how the food environment influences dietary behavior [21,71]. In a recent study, those who reported eating more fruits and vegetables each week were more likely to order their groceries online [72]. These findings imply that financial incentive programs focusing on fruits and vegetables may be appealing and successful ways to promote healthy purchasing practices among SNAP participants while ordering groceries online. Our results are consistent with previous studies [72,73] that younger adults are most likely to order groceries online, possibly related to their comfort with technology. Still, more efforts are needed to address barriers that food assistance recipients face and to improve both digital and nutrition literacy [21]. For example, according to an online survey study assessing reasons for discontinuing participation in food assistance programs, households receiving WIC were particularly discouraged by difficulties in redeeming benefits, and SNAP participants expressed a desire for better online grocery purchasing experiences [74]. In the present study, the main obstacles to online grocery shopping were a need for more knowledge, expenses not covered by food assistance programs, and reservations about food selection. Although there has been an increase in studies on using SNAP benefits as a payment method for online groceries, more evidence is needed to understand the potential effect on diet quality.

The present study has a number of limitations and strengths. The use of a convenience sample technique is a constraint of the current study since there is a risk of either over- or under-representation of population groups. Our sample’s demographic characteristics, however, are closely aligned with those of the target zip codes. Likewise, we used self-reported data, in which survey respondents might be more prone to underreporting. As a result of our cross-sectional design, cause and effect could not be elucidated. Using an online survey may have introduced sampling bias in favor of individuals familiar with digital equipment and internet access. Although we performed an adjusted analysis to control factors previously associated with diet quality, unmeasured confounding variables could affect food assistance participation and perceived food environment status. Overall, our confidence that the patterns we found may reflect interactions of the perceived food environment, food assistance participation, and online grocery ordering on diet quality is strengthened by the robustness of our results across two measures of diet quality.

## 5. Conclusions

This study is one of the first to examine associations between online grocery ordering and diet quality among individuals from a low-income and low food access community, underlining links between the perception of the food environment, food assistance, and online ordering. Adults who did not order groceries online and perceived their store and home environment to be the least healthful had the poorest diet quality. Given the potential role of online grocery ordering in addressing food accessibility for underprivileged and vulnerable populations in the United States, this research brings encouraging findings that support online grocery shopping as an important step toward addressing diet quality in these demographics. However, greater effort is needed to understand the perceptions of the food environment and online ordering experiences among participants of food assistance programs to design the most effective interventions.

## Figures and Tables

**Table 1:** Sociodemographic, lifestyle, and health characteristics of adults who live in a community of low-income and low-access to healthy food community in Northeast Connecticut by reported use of ordering groceries online.

Characteristic	Total (n = 276) % or M (SD)	In Person (n = 153) % or M (SD)	Online (n = 123) % or M (SD)	*p* Value
**Age**	35.2 ± 12.04	36.5 ± 12.85	33.6 ± 10.79	**0.051**
**Gender**				**0.014**
Men	25.4 (70)	10.9 (30)	14.5 (40)	
Women	74.6 (206)	44.6 (123)	30.1 (83)	
**Race and ethnicity**				**0.016**
White/Caucasian	52.2 (144)	26.1 (72)	26.1 (72)	
Latino/Hispanic	37.0 (102)	24.3 (67)	12.7 (35)	
Black/African American	5.8 (16)	1.8 (5)	4.0 (11)	
Others ^1^	5.1 (14)	3.3 (9)	1.8 (5)	
**Birthplace**				**<0.001**
Connecticut (CT)	62.7 (173)	28.6 (79)	34.1 (94)	
U.S. outside CT	30.8 (85)	21.4 (59)	9.4 (26)	
Another country	6.5 (18)	5.4 (15)	1.1 (3)	
**Primary language ^2^**				**0.028**
English	89.5 (247)	47.5 (131)	42.0 (116)	
Non-English	10.5 (5.7)	8.0 (14.4)	2.5 (7)	
**Household size**				0.254
Adults only	35.9 (99)	21.0 (58)	14.9 (41)	
Households with children	64.1 (177)	34.4 (95)	29.7(82)	
**Years at current address**				0.372
<1 year	14.9 (41)	9.8 (27)	5.1 (14)	
≥1 year, <2 years	13.4 (37)	8.3 (23)	5.1 (14)	
≥2 years, <5 years	28.3 (78)	15.2 (42)	13.0 (36)	
≥5 years, <10 years	15.6 (43)	8.7 (24)	6.9 (19)	
≥10 years	27.9 (77)	13.4 (37)	14.5 (40)	
**Marital status**				0.480
Married/partner	48.2 (133)	26.1 (72)	22.1 (61)	
Separated/divorced/widowed	13.4 (37)	6.5 (18)	6.9 (19)	
Never married	38.4 (106)	22.8 (63)	15.6 (43)	
**Education**				0.243
≤8th grade/Some High School	10.9 (30)	5.1 (14)	5.8 (16)	
H.S. graduate/GED	26.8 (74)	17.4 (48)	9.4 (26)	
Some college or technical	33.7 (93)	17.4 (48)	16.3 (45)	
Graduate/professional degree	28.6 (79)	15.6 (43)	13.0 (36)	
**Employment**				0.311
Full-time	47.8 (132)	25.0 (69)	22.8 (63)	
Part-time employment	18.8 (52)	9.4 (26)	9.4 (26)	
Unemployed, active seeking	11.6 (32)	6.9 (19)	4.7 (13)	
Unemployed, not seeking ^3^	21.7 (60)	14.1 (39)	7.6 (21)	
**Health Insurance**				0.674
Public (Federal/State)	63.8 (176)	4.0 (95)	2.2 (81)	
Private	30.1 (83)	34.4 (47)	29.3 (36)	
None	6.2 (17)	17.0 (11)	13.0 (6)	
**Body Size Category ^4^**				0.529
Normal weight	29.8 (82)	17.8 (49)	12.0 (33)	
Overweight	46.9 (129)	24.4 (67)	22.5(62)	
Obesity	23.3 (64)	13.1 (36)	10.2 (28)	
**Self-rated Health Status**				0.973
Poor/Fair	31.5 (87)	17.8 (49)	13.8 (38)	
Good	47.8 (132)	26.4 (73)	21.4 (59)	
Very good/Excellent	20.7 (57)	11.2 (31)	9.4 (26)	
**Self-rated Diet Status ^5^**				**0.028**
Poor/Fair	47.5 (131)	27.9 (77)	19.6 (54)	
Good	39.1 (108)	22.8 (63)	16.3 (45)	
Very good/Excellent	13.4 (37)	4.7 (13)	8.7 (24)	
**Food security status ^6^**				0.445
Food secure	82.6 (228)	44.9 (124)	37.7 (104)	
Food insecure	17.4 (48)	10.5 (29)	6.9 (19)	
**# of drivable vehicles ^7^**				0.222
0 vehicles	8.0 (22)	3.6 (10)	4.4 (12)	
≥1 vehicle	92.0 (252)	51.8 (142)	40.1 (110)	
**Food Assist. Participation ^8^**				**0.001**
1 program	27.5 (76)	15.6 (43)	12.0 (33)	
2 or 3 programs	32.2 (89)	13.0 (36)	19.2 (53)	
None	40.2 (111)	26.8 (74)	13.4 (37)	
**Cigarette smoking**				**0.047**
Current (inc e-cigarettes)	23.9 (66)	10.1 (28)	13.8 (38)	
Former	15.9 (44)	9.1 (25)	6.9 (19)	
Never	60.1 (166)	36.2 (100)	23.9 (66)	
**Physical Activity/week**				0.331
<150 min	85.5 (236)	46.4 (128)	39.1 (108)	
≥150 min	14.5 (40)	9.1 (25)	5.4 (15)	

^1^ Includes Asian/South Asian/Pacific Islander, American Indian or Alaskan Native, or multi-racial. ^2^ Included those who selected to take the survey in Spanish. ^3^ Includes students, retirees, homemakers, disabled, etc. ^4^ Assessed by Stunkard’s figure rating scale [37], (1–2 = underweight, 3–4 = normal weight, 5–6 = overweight, and 7–9 = obese) (2 missing data). ^5^ Assessed by [40]. ^6^ Assessed by 2-item food security interview [41]. ^7^ In response to the question, How many drivable motor vehicles (cars, trucks, and motorcycles) are there in your household? ^8^ Includes Supplemental Nutrition Assistance Program (SNAP), Special Supplemental Nutrition Program for Women, Infants, and Children (WIC), Child and Adult Care Food Program (CACFP), food pantry, and mobile food trucks.

**Table 2:** Perceived store consumer, community, and home food environments among adults (n = 276) living in a low-income, low food access community in Northeast Connecticut [10,42].

Composite Item	Survey Item	Item Range	Number of Items	Total Possible Range	Scores of Study Sample
Store consumer Food Environment					Mean ± SD
Quality of fruits and vegetables in neighborhood	Please mark whether you agree or disagree with the following statements: – The fresh produce in my neighborhood is of high quality.	1–5 ^a^	1	1–5	3.55 ± 0.97
Large selection of fruits and vegetables in neighborhood	Please mark whether you agree or disagree with the following statements: – There is a large selection of fresh fruits and vegetables in my neighborhood.	1–5 ^a^	1	1–5	3.71 ± 1.01
Ease of purchase of fruits and vegetables in neighborhood	Please mark whether you agree or disagree with the following statements: – It is easy to buy fruits and vegetables in my neighborhood.	1–5 ^a^	1	1–5	3.88 ± 1.00
Ease of purchase of fruits and vegetables online	Please mark whether you agree or disagree with the following statements: – It is easy to buy fruits and vegetables through online shopping.	1–5 ^a^	1	1–5	3.10 ± 1.05
Ease to buy low-fat products in neighborhood	Please mark whether you agree or disagree with the following statements: – It is easy to buy low-fat products, such as low-fat milk or lean meats, in my neighborhood.	1–5 ^a^	1	1–5	3.87 ± 0.86
Sweetened beverages, snack foods (chips), sweets are easy to obtain in my neighborhood	Please mark whether you agree or disagree with the following statements: – It is easy to buy sweetened beverages, snack foods (chips), sweets are easy to obtain in my neighborhood.	1–5 ^a^	1	1–5	4.13 ± 0.92
There is a large selection of sweetened beverages, snack foods (chips), sweets in my neighborhood.	Please mark whether you agree or disagree with the following statements: – There is a large selection of sweetened beverages, snack foods (chips), sweets in my neighborhood.	1–5 ^a^	1	1–5	4.13 ± 0.94
**Store healthy available**	(Quality, Availability, Ease of Healthy fruits and vegetables and low-fat products)—(Ease and Selection of Unhealthy) (alpha = 0.882)	1–5	6	−3–16	9.86 ± 3.11
**Store price produce**	At the store where you buy most of your food, how would you rate the price of fresh fruits and vegetables?	1–4 ^b^	1	1–4	2.37 ± 0.81
**Community food environment**					
Near or on the way to other places	How important in your decision to shop at the store where you buy most of your food… Near or on the way to other places where you spend time	1–4 ^e^	1	1–4	2.72 ± 0.92
Access to public transportation	How important in your decision to shop at the store where you buy most of your food… Access to public transportation	1–4 ^e^	1	1–4	2.16 ± 1.17
Near your home	How important in your decision to shop at the store where you buy most of your food… Near your home	2–4 ^e^	1	2–4	3.02 ± 0.80
**Store access**	(Store near others, access to public transportation, and store near home) (alpha = 0.47)	1–4	3	3–12	7.87 ± 2.07
**Home food environment**					
Availability of fruits and vegetables in the home	Please indicate whether each of these food items were available in your home in the past week: Bananas, Apples, Canned/Frozen Fruits, Canned/Frozen Vegetables, Carrots, Tomatoes, Dark leafy greens (spinach, collards, kale, etc.)	0–1	7	0–7	4.26 ± 1.86
Availability of healthy food in the home	Please indicate whether each of these food items were available in your home in the past week: Bananas, Apples, Canned/Frozen Fruits, Canned/Frozen Vegetables, Carrots, Tomatoes, Dark leafy greens (spinach, collards, kale, etc.), Beans/Lentils, Whole Grain Bread, Brown Rice, Low-Fat Milk	0–1	11	0–11	6.07 ± 2.69
Availability of unhealthy food in the home	Please indicate whether each of these food items were available in your home in the past week: Candy or cookies, snack chips (potato chips, corn chips, tortilla chips, etc.), regular whole milk, regular (non-diet) soda, regular hot dogs, white bread, white rice	0–1	7	0–7	4.04 ± 1.83
Accessibility of healthy food in the home	In your home, how often do you… – Have fruits and vegetables in the refrigerator? – Have fruit available in a bowl or on the counter?	1–4 ^c^	2	1–4 ^d^	3.24 ± 0.77
Accessibility of unhealthy food in the home	In your home, how often do you… – Have candy or chips available to eat? – Have ice cream, cake, pastries, or ready-to-eat sweet baked goods (cookies, brownies, etc.)?	1–4 ^c^	2	1–4 ^d^	2.77 ± 0.73
**Average Home Food Environment**	(Healthy Food Available & Access)—(Unhealthy Food Available & Access) (alpha = 0.732)		5	−3.5–19	6.77 ± 4.62

^a^ Response options: 1 = strongly disagree to 3 = neither agree nor disagree to 5 = strongly agree. ^b^ Response options: 1 = very expensive, 2 = somewhat expensive, 3 = not expensive, 4 = very inexpensive. ^c^ Response options: 1 = never or rarely to 4 = almost always. ^d^ an average was taken across items. ^e^ Importance rated as =not at all, 2 = a little, 3 = somewhat, 4 = very.

**Table 3:** Frequency of participants (n = 276) who meet the U.S. Dietary Guidelines [43] food group or component recommendations *.

	Fruit, Any Kind	Whole Fruit	Vegetable, Any Kind	Beans, Peas, Lentils	Whole Grains	Milk, Any Type	Low Fat Dairy	Seafood	Nuts/Seeds	Sugar-Sweetened Beverages
**Recommendations**	2 cups/d	1 cup/d	2.5 cups/d	1.5 cups/wk	3 oz/d	3 cups/d	3 cups/d	8 oz/wk	5 oz/wk	≤12 oz/d
**Meeting Dietary Guidelines**	**116 (42%)**	**106** **(38%)**	**69** **(25%)**	**176 (64%)**	**69** **(25%)**	**59** **(21%)**	**46 (17%)**	**113 (41%)**	**175 (63%)**	**136** **(49%)**

* Recommendations including daily fruit or 100% fruit juice, whole fruit, vegetable, beans, whole grains, milk of any type, low-fat dairy, and limited consumption of sugary drinks.

**Table 4:** Unadjusted measures of diet quality by sociodemographic and health characteristics among samples of adults living in a community with low access to healthy foods in Northeast CT (*N* = 276).

	N	sHEI Score		Liking-Based DQI ^7^	
Characteristic		Mean	*p* Value	Mean	*p* Value
**Gender**			**0.001**		0.114
Men	25.4 (70)	39.50		−8.14	
Women	74.6 (206)	44.01		−0.61	
**Race**			0.721		0.535
White/Caucasian	52.2 (144)	42.49		−3.49	
Latino/Hispanic	37.0 (102)	43.01		−2.01	
Black/African American	5.8 (16)	45.59		8.25	
Others ^1^	5.1 (14)	42.56		−8.86	
**Birthplace**			0.309		0.522
Connecticut (CT)	62.8 (173)	43.54		−1.35	
U.S. outside CT	30.8 (85)	41.45		−5.83	
Another country	6.5 (18)	43.05		2.13	
**Primary language ^2^**			0.360		**<0.001**
English	89.5 (247)	43.06		−4.86	
Non-English	10.5 (5.7)	41.21		18.69	
**Household size**			0.169		0.349
Adults only	35.9 (99)	41.72		−5.10	
Households with children	64.1 (177)	43.51		−1.06	
**Years at current address**			0.101		0.161
<1 year	14.9 (41)	40.97		−3.67	
≥1 year, <2 years	13.4 (37)	44.38		−4.80	
≥2 years, <5 years	28.3 (78)	44.52		−8.45	
≥5 years, <10 years	15.6 (43)	39.92		−2.77	
≥10 years	27.9 (77)	43.12		5.21	
**Marital status**			0.784		0.544
Married/partner	48.2 (133)	43.10		−3.87	
Separated/divorced/widowed	13.4 (37)	41.78		3.10	
Never married	38.4 (106)	42.96		−2.81	
**Education**			0.784		**0.026**
≤8th grade/Some High School	10.9 (30)	44.87		−3.52	
H.S. graduate/GED	26.8 (74)	41.25		−12.60	
Some college or technical	33.7 (93)	42.54		2.02	
Graduate/professional degree	28.5 (79)	44.00		1.78	
**Employment**			0.263		0.826
Full-time	47.8 (132)	43.12		−1.96	
Part-time employment	18.8 (52)	44.75		−6.47	
Unemployed, active seeking	11.6 (32)	44.21		−0.59	
Unemployed, not seeking **^3^**	21.7 (60)	39.95		−1.28	
**Health Insurance**			0.832		0.767
Public (Federal/State)	63.8 (176)	41.66		−0.63	
Private	30.1 (83)	42.79		−1.62	
None	6.2 (17)	43.27		−4.78	
**Body Size Category ^4^**			**0.019**		0.853
Normal weight	29.8 (82)	45.56		−0.68	
Overweight	46.9 (129)	41.74		−3.35	
Obesity	23.3 (64)	41.75		−2.96	
**Self-rated Health Status**			**0.016**		**0.022**
Poor/Fair	31.5 (87)	41.05		−9.92	
Good	47.8 (132)	42.68		−1.21	
Very good/Excellent	20.7 (57)	46.07		5.76	
**Self-rated Diet Status**			**<0.001**		**<0.001**
Poor/Fair	47.5 (131)	39.02		−11.82	
Good	39.1 (108)	45.82		5.54	
Very good/Excellent	13.4 (37)	47.88		6.40	
**Food security status ^5^**			0.345		0.744
Food secure	82.6 (228)	42.60		−2.82	
Food insecure	17.4 (48)	44.15		−1.05	
**# Drivable vehicles**			0.176		0.738
0 vehicles	8.0 (22)	45.68		−0.53	
≥1 vehicle	92.0 (252)	42.57		−3.09	
**Food Assist. Participation ^6^**			**0.021**		0.841
1 program	27.5 (76)	41.44		−0.72	
2 or 3 programs	32.2 (89)	45.35		−2.44	
None	40.2 (111)	41.86		−3.75	
**Online Grocery Ordering**			**0.024**		0.056
Yes	44.6 (123)	44.42		1.87	
No	55.4 (153)	41.62		−6.06	
**Cigarette smoking**			0.352		0.131
Current (inc e-cigarettes)	23.9 (66)	41.48		−9.17	
Former	15.9 (44)	44.31		3.52	
Never	60.1 (166)	43.04		−1.39	
**Physical Activity/week**			**0.006**		**0.009**
<150 min	85.5 (236)	42.16		−4.75	
≥150 min	14.5 (40)	47.04		10.40	

^1^ Includes Asian/South Asian/Pacific Islander, American Indian or Alaskan Native, or multi-racial. ^2^ Included those who selected to take the survey in Spanish. ^3^ Include student, retired, home-maker, disabled, etc. ^4^ Assessed by Stunkard’s figure rating scale [37], normal weight (1–2 = underweight, 3–4 = normal weight, 5–6 = overweight, and 7–9 = obese) (2 missing data). ^5^ Assessed by 2-item food security interview [41]. ^6^ Includes Child and Adult Care Food Program (CACFP), food pantry, and mobile food trucks. ^7^ The liking-based Diet Quality Index (DQI) assessed by survey-reported food likes/dislikes and conceptual food groups that were conceptually weight following the Dietary Guidelines 2020 and averaged [43].

**Table 5:** Main and moderating effects of food assistance participation and ordering online on measures of diet quality, adjusted for covariates among samples of adults living in a community of low access to healthy foods in Northeast CT (*N* = 276).

		sHEI Score		Liking-Based DQI ^6^		
	Partial	95% CI	*p*-Value	Partial	95% CI	*p*-Value
**Crude linear models**						
*Store consumer food environment*						
Store healthy available	0.089	−0.01–0.7	0.140	0.037	−0.9–1.7	0.541
Store price produce	−0.043	−2.1–1.0	0.477	−0.148	−11.2– −1.2	**0.015**
*Community food environment*						
Store access	0.013	−0.5–0.7	0.824	0.057	−1.0–2.9	0.352
*Home food environment*						
Average Home Food Environment	0.293	0.4–0.9	**<0.001**	0.354	1.8–3.4	**<0.001**
**Adjust Linear Model 1 †**						
*Store consumer food environment*						
Store healthy available	0.076	−0.1–0.6	0.211	0.007	−1.2–1.3	0.906
Store price produce	−0.050	−2.1–0.9	0.412	−0.111	−9.3–0.4	0.072
*Community food environment*						
Store access	−0.012	−0.7–0.5	0.851	0.047	−1.2–2.6	0.451
*Home food environment*						
Average Home Food Environment	0.308	0.4–1.0	**<0.001**	0.322	1.5–3.1	**<0.001**
**Adjust Linear Model 2 ††**						
*Store consumer food environment ¥¥*						
Store healthy available	0.051	−0.2–0.6	0.408	−0.037	−1.7–0.9	0.551
Store price produce	−0.034	−1.9–1.1	0.584	−0.103	−8.8–0.7	0.098
*Community food environment ¥¥*						
Store access	−0.011	−0.6–0.5	0.853	0.052	−1.1–2.7	0.405
*Home food environment ††*						
Average Home Food Environment	0.290	0.4–0.9	**<0.001**	0.303	1.3–3.0	**<0.001**
**Interaction Model 3 ¥**						
Store healthy available * Fd Ass	0.10	0.0–0.3	0.11	0.06	−0.2–0.7	0.338
Store price produce * Fd Ass	0.046	−0.4–0.8	0.459	−0.01	−1.9–1.8	0.935
Store access * Fd Ass	0.048	−0.1–0.2	0.433	0.043	−0.4–0.7	0.496
Average Home Food Environment * Fd Ass	0.192	0.1–0.4	**0.002**	0.226	0.4–1.4	**<0.001**
**Interaction Model 4 ¥¥**						
Store healthy available * Grocery On	0.144	0.0–0.5	**0.019**	0.128	0.1–1.5	**0.024**
Store price produce * Grocery On	0.114	0.0–1.8	0.063	0.140	0.5–6.6	**0.024**
Store access * Grocery On	0.099	−0.1–0.5	0.106	0.158	0.3–2.1	**0.011**
Average Home Food Environment * Grocery On	0.178	0.1–0.7	**0.004**	0.203	0.6–2.3	**<0.001**

† Model 1 Adjusted for age (log transformed), sex (m/f), language (English, non-English), education (Some high school or less, High school graduate/GED, Some college/technical, Graduate/prof degree), household with children (y/n), ordering groceries online (y/n). †† Model 2 adds Physically active (<150 min per week, ≥150 min per week), Body Size Category (normal weight, overweight and obesity), Perceived Health (poor/fair, good, very good/excellent), and Smoking Status (current (including e-cigarettes), former, never) to model 1. ¥ Model 3 includes adjustment for variables in Models 1 and Model 2 and interaction with participation in food assistance (0 = none, 1 = 1 program, 2 ≥ programs). ¥¥ Model 4 includes adjustment for variables in Models 1 and Model 2 and interaction with ordering groceries online (0 = no, 1 = yes). ^6^ The liking-based Diet Quality Index (DQI) assessed by survey-reported food likes/dislikes and conceptual food groups that were conceptually weight following the Dietary Guidelines 2020 and averaged [43]

## Data Availability

The data presented in this study are available on request from the corresponding authors.

## Appendix A

Food groups in the Liking Survey [35] with mean and standard deviation.

	**Mean**	**SD**
Fruits (Bananas, Orange Juice, Pineapple)	54.05	30.58
Refined Carbohydrates (White Rice, Rolls, Pasta)	50.72	32.35
High Fat (Salty Snacks, French Fries, Fried Chicken or Fish, Bacon, Fast Food)	45.30	29.47
Healthy Fats (Nuts, Olive Oil)	44.72	27.37
High Fat Protein (Cheddar Cheese, Whole Milk, Pizza, Beef Steak)	42.30	28.63
Sweet (Ice Cream, Cookies, Soda, Coffee Drinks)	40.72	32.92
Fiber (Whole wheat bread, Beans, Tortilla, Oatmeal)	38.12	29.83
Low-Fat Protein (Tuna, Baked Chicken, Pork Chops)	35.96	35.85
Vegetables (Broccoli, Carrots, Greens, Tomato)	34.15	39.86
Liking-based Diet Quality Index	−1.66	32.58

## Appendix B

Food groups and components of the sHEI survey [31] that are used to calculate the sHEI.

**Dietary Component**	**Range**	**Mean ± SD**
Total Fruits	0 to 5	1.61 ± 2.34
Whole Fruit	0 to 5	3.02 ± 1.67
Total Vegetables	0 to 5	2.66 ± 0.77
Greens and Beans	0 to 5	3.19 ± 2.41
Whole Grains	0 to 10	4.01 ± 2.00
Dairy	0 to 10	4.12 ± 1.42
Total Protein Foods	0 to 5	4.57 ± 1.30
Seafood and Plant Protein	0 to 5	2.33 ± 1.46
Fatty Acid Ratio	0 to 10	0.63 ± 1.68
Refined Grains	0 to 10	6.32 ± 3.22
Sodium	0 to 10	4.00 ± 2.47
Added Sugars	0 to 10	3.41 ± 3.77
Saturated Fats Component	0 to 10	3.00 ± 1.53
Total Diet Quality Score	0 to 100	42.87 ± 10.32
